# Orbital Interaction and Electron Density Transfer in Pd^II^([9]aneB_2_A)L_2_ Complexes: Theoretical Approaches

**DOI:** 10.3390/molecules181012687

**Published:** 2013-10-14

**Authors:** Ock Keum Kwak, Mahreen Arooj, Yong-Jin Yoon, Euh Duck Jeong, Jong Keun Park

**Affiliations:** 1 Department of Chemistry Education and Research Institute of Natural Science, Educational Research Institute Teachers College, Gyeongsang National University, Jinju 660-701, Korea; 2 Department of Chemistry and Research Institute of Natural Science, Gyeongsang National University, Jinju 660-701, Korea; 3 Division of High-Technology Materials Research, Korea Basic Science Institute, Busan 618-230, Korea

**Keywords:** macrocyclic tridentate, position selectivity, steric and electronic effects, orbital interaction, geometric configuration

## Abstract

The geometric structures of Pd-complexes {Pd([9]ane**B**_2_**A**)**L**_2_ and Pd([9]ane**BAB**)**L**_2_ where **A** = P, S; **B** = N; **L** = PH_3_, P(CH_3_)_3_, Cl^−^}, their selective orbital interaction towards equatorial or axial (soft **A**…Pd) coordination of macrocyclic [9]ane**B**_2_**A** tridentate to Pd**L**_2_, and electron density transfer from the electron-rich *trans* **L**-ligand to the low-lying unfilled a_1g_(5s)-orbital of Pd**L**_2_ were investigated using B3P86/lanl2DZ for Pd and 6-311+G** for other atoms. The pentacoordinate *endo*-[Pd([9]ane**B**_2_**A**)(**L**-donor)_2_]^2+^ complex with an axial (soft **A**--Pd) quasi-bond was optimized for stability. The fifth (soft **A**--Pd) quasi-bond between the σ-donor of soft **A** and the partially unfilled a_1g_(5s)-orbital of Pd**L**_2_ was formed. The pentacoordinate *endo*-Pd([9]ane**B**_2_**A**)(**L**-donor)_2_]^2+^ complex has been found to be more stable than the corresponding tetracoordinate *endo*-Pd complexes. Except for the *endo*-Pd pentacoordinates, the tetracoordinate Pd([9]ane**BAB**)**L**_2_ complex with one equatorial (soft **A**-Pd) bond is found to be more stable than the Pd([9]ane**B**_2_**A**)**L**_2_ isomer without the equatorial (**A**-Pd) bond. In particular, the geometric configuration of *endo-*[Pd([9]ane**PNP**)(**L**-donor)_2_]^2+^ could not be optimized.

## 1. Introduction

Carbon-carbon/carbon-heteroatom (e.g., C–C, C–N, C–O, C–S) bond formation using palladium-mediated cross-coupling reactions has been extensively studied during the last few decades [1,2,3,4,5,6,7,8,9,10,11,12,13,14,15,16,17,18,19,20,21,22,23,24,25,26,27,28,29,30,31,32,33,34,35,36]. In this context, the achievements in development of novel and efficient synthetic methodologies for these types of reactions have been acknowledged with the 2010 Nobel Prize in Chemistry. Much attention has been paid to the design and development of powerful Pd-catalysts/precursors [1,2,3,4,5,6,7,8,9,10,11,12,13,14,15,16,17,37,38,39,40,41,42,43] that contain bulky and electron-rich ligands (such as phosphines and *N*-heterocyclic carbenes, NHCs) and to their potential synthetic applications [18,19,20,21,22,23,24,25,26,27,28,29,30,31,32,33,34,35,36] in the Pd-catalyzed cross-couplings of various substrates (such as allyl, aryl, and vinyl halides and olefins). Catalytic activity enhancement of Pd^(0)^(**L**)_n_-precursors is greatly influenced by the electronic and steric properties of electron-rich and bulky **L-**ligands [1,2,3,4,5,6,7,8,18,19,20,21,22,23,24,25,26,27,28,29,30,31,32,33,34,35,36,37,38,39,40,41,42]. Due to the electronic effect of the *trans* **L-**donor, the oxidation state of Pd in neutral and anionic Pd^(0)^**L**_n_X catalysts is zero. The geometric structure of bulky **L-**ligand affects the oxidative addition reaction of Ar-X to the Pd^(0)^(**L**)_n_-precursor [6,7,8,25,26,27,28,29,30,31,32].

The geometric structures of the active RPd(**L**)_n_X intermediates produced in various steps of Pd-mediated cross-coupling reactions (e.g., oxidative addition, transmetalation, reductive elimination) have been investigated both theoretically [9,10,11,12,13,14,15,16,17] and experimentally [18,19,20,21,22,23,24,25,26,27,28,29,30,31,32,33,34,35,36,37,38,39,40,41,42]. The oxidative addition of substrates (R-X) to Pd**L**_n_-precursor resulted in penta-, hexa-, and octacoordinate geometries [33,34,35,36] of the RPd^II^(**L**)_n_X intermediates with relative stability and a life time of 30 s [3]. The binding atoms in the penta-, hexa-, and octacoordinate Pd intermediates are not located on the x, y, and z-axis of trigonal bipyramidal, octahedral, and cubic structures. In particular, the geometric conformation of RPd(**L**)_n_X intermediates is altered by the *cis-trans* isomerization of **L**-ligand. The isomerization has been exclusively explained by the energy relationship between the isomers and low potential barrier (or binding energy of Pd-**L**) [3,4,5,6,7,8,44,45]. However, Goossen *et al*. [14,15] provided no evidence for mechanistic steps involving stable pentacoordinate Pd^II^ intermediates in Pd-mediated cross-coupling reactions. To the best of our knowledge, the relative stabilities of various RPd(**L**)_n_X intermediates produced by oxidative addition of the substrate (R-X) to Pd**L**_n_ and the geometric changes in intramolecular interaction of RPd^II^(**L**)_n_X have not been investigated. 

In previous studies using hemilabile multidentate ligands [37,38,39,40,41,42,43,44,45,46,47], exceptional oxidation state and specific coordination selectivity have been observed for the Pd([9]ane**B**_2_**A**)**L**_2_ complexes with mixed soft **A** and hard **B** tridentates. The uncommon geometric structures of Pd([9]ane**B**_2_**A**)**L**_2_ complexes with an axial (**A**…Pd) interaction were mainly formed under the following restrictive conditions: (1) coordination bonds of **P**- and **N**-functionalized derivatives are present [18,19,20,21,22,23,24,25,26,27,28,29,30,31,32,33,34,35,36,37,38,39] and (2) polymeric side chain interactions exist [40,41,42]. In the Pd complex with an apical (hard **N**…Pd) interaction [42], the apical interaction was explained by an antibonding interaction between the lone pair of the apical **N** site and the d_z2_-orbital of the Pd^II^ species. In the ligand exchange reaction of square-planar Pd complexes, a vertical **L**…Pd interaction also has been optimized [43,44,45,46,47]. The mechanism of hydration exchange processes in the five-coordinate Pd^II^ intermediates suggested two models for the axial interaction: σ-donor…d_z_^2^-orbital and HOH…d_z_^2^-orbital. The apical (**A**…Pd) orbital interaction of Pd([9]ane**B**_2_**A**)**L**_2_ complex in mixed **A** and **B** sites has not been explained very well. 

**Scheme 1:**
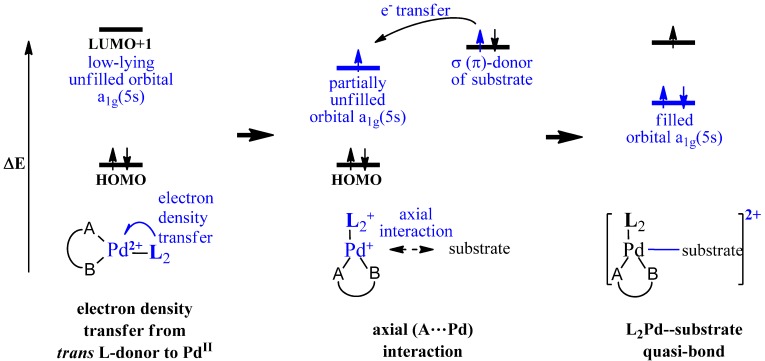
Two-step mechanism for the electron density transfer from an equatorial *trans* **L**-donor {PH_3_, P(CH_3_)_3_} and a σ-donor of substrate to a low-lying unoccupied a_1g_(5s)-orbital of the Pd^II^ center.

Origins of the unusual coordination structures for the Pd complexes and the configurational changes in the ArPdL_n_X intermediates of Pd-mediated cross-coupling reaction have not been explored yet. To investigate these properties, the present study proposed the following geometric structures and the relative stabilities of macrocyclic Pd([9]ane**B**_2_**A**)**L**_2_ complexes within the frameworks of its orbital interaction and electronic effect. For the Pd-mediated cross-coupling reactions, we suggest a two-step mechanism for the electron density transfer: the abundant electron density of the *trans* **L**-donor may transfer to a low-lying unoccupied a_1g_(5s)-orbital of Pd^II^ and then the partially unfilled a_1g_(5s)-orbital can interact with the Lewis base substrate (σ-donor) as shown in Scheme 1.

**Scheme 2:**
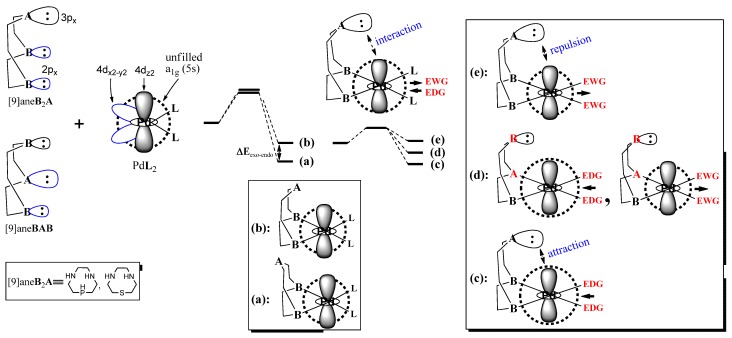
A plausible orbital interaction of an equatorial or axial coordination bond of [9]ane**B**_2_**A** (or [9]ane**BAB**) with Pd**L**_2_. (**a**): *exo*-type with an apical site pointing to outside. (**b**): *endo*-type with an apical site pointing to the Pd center. (**c**): *endo*-Pd complex with an apical (**A**…Pd) attraction. (**d**): *endo*-Pd complex without any axial interaction. (**e**): *endo*-Pd complex with an apical (**A**…Pd) repulsion.

To justify the intermediate step for the additional reaction of [9]ane**B**_2_**A** to Pd**L**_2_, the relative stabilities (Δ _exo-endo_, Δ_BAB-B2A_) and the apical and equatorial orbital interactions [σ-donor…a_1g_(5s), σ-donor…4d_x2-y2_] shown in Scheme 2 are suggested in this research exertion. The relative affinity of Pd towards the soft **A** (or hard **B**) in Pd**L**_2_, the electronic characteristics owing to the ***donating*** (or ***withdrawing***) property of the *trans* **L-**ligand, the axial σ-donor…a_1g_(5s) interaction between the σ-donor of the **A** site and the low-lying unfilled a_1g_(5s)-orbital of Pd^II^L_2_, and the relative stability of the uncommon Pd complexes were examined in detail.

## 2. Computational Methods

To explore the geometric structure and the relative stability of the Pd complexes, we selected the Pd([9]ane**B**_2_**A**)**L**_2_ [**A** = P, S; **B** = N; **L** = donor {PH_3_, P(CH_3_)_3_}, acceptor (Cl^−^)] complex as a model with [9]ane**B**_2_**A**, PH_3_, P(CH_3_)_3_, and Cl^−^ groups as ligands. The equilibrium structure of tetracoordinate Pd complexes was fully optimized with the B3P86/6-311+G** (lanl2DZ for Pd) level using Gaussian 03 [48]. The macrocyclic [9]ane**N**_2_**P** and [9]ane**N**_2_**S** ligands are 1,4-diaza-7-phospacyclononane and 1,4-diaza-7-thiacyclononane, respectively (as shown in Scheme 2). The hybrid B3P86 density functional utilizes the exchange function of Becke [49,50] in conjunction with the Perdew 1986 correlation function [51] and yields good structural and energetic information, even for relatively large chemical systems such as transition metal complexes. For the optimized equilibrium Pd^II^ complexes, the atomic charges obtained by the CHelpG method were analyzed using atomic radii (H = 0.53 Å, C = 0.67 Å, N = 0.56 Å, O = 0.48 Å, P = 0.98 Å, S = 0.88 Å, Cl^−^ = 1.67 Å, Pd^II^ = 0.78 Å) [52]. The relative energies of the Pd^II^ complexes were compared. To confirm the existence of stable structures, the harmonic vibration frequencies of the species were analyzed at the B3P86 level. In addition, the geometric structures of *endo*-Pd([9]ane**A**_2_**B**)**L**_2_ {**A** = P, S; **B** = N; **L** = PH_3_, P(CH_3_)_3_, Cl^−^} were optimized at the B3P86/6-311+G** (lanl2DZ for Pd) level. The optimized structures including HOMO and the geometric parameters are described in Supplementary Information Figure S1 and Table S1, respectively. To check the rationality of our results at the effective core potential level for Pd, the geometric structures of the equilibrium Pd complexes were also optimized at the CAM-B3LYP//6-311+G** (lanl2DZ for Pd) and B3P86/6-311+G** (3-21g* for Pd) levels using Gaussian 09. Further, optimized structures and parameters are given in Supplementary Information Figures S2 and S3 and Tables S2 and S3.

## 3. Results and Discussion

**Figure 1:**
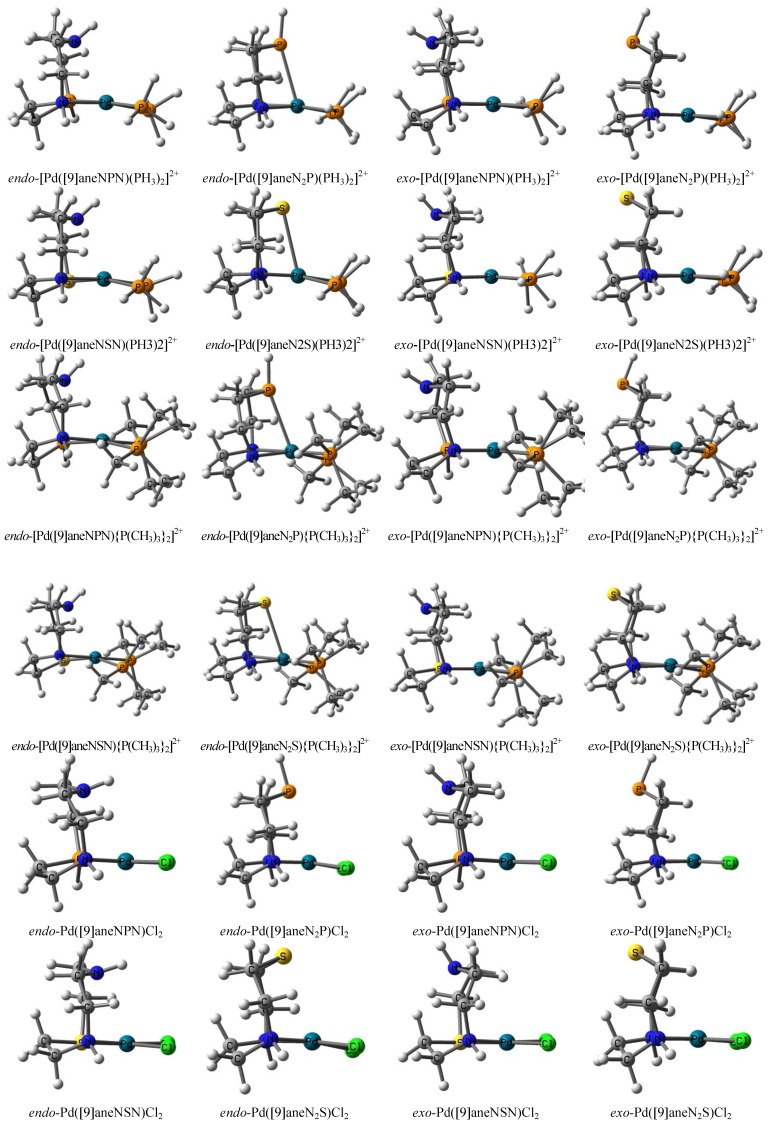
The geometric structures of the Pd complexes [Pd([9]ane**B**_2_**A**)**L**_2_ and Pd([9]ane**BAB**)**L**_2_ (**A** = P, S; **B** = N; **L** = PH_3_, P(CH_3_)_3_, Cl^−^] were optimized at the B3P86/6-311+G^**^ (lanl2DZ for Pd) level. All structures are viewed from the side.

Optimized geometric structures of Pd([9]ane**B**_2_**A**)**L**_2_ and Pd([9]ane**BAB**)**L**_2_ types are represented in Figure 1, and their parameters are listed in Table 1. The values of optimized parameters in this study were compared to the values reported previously [17,18,23,37,40,41,42,44,53,54]. In the Pd complexes, the tetracoordinate structures of both the *endo*- and *exo*-types of structures were optimized. Pd^II^ locates at the center of the tetracoordinate mean plane that is coordinated with a [9]ane**B**_2_**A** (or [9]ane**BAB**) tridentate and two monodentate ligands. In the case of the *endo*-[Pd([9]ane**B**_2_**A**)(**L**-donor)_2_]^2+^ with *trans* **L**-donor, *endo*-[Pd([9]ane**B**_2_**A**)(**L**-donor)_2_]^2+^ pentacoordinates with an axial (**A**--Pd) quasi-bond were stably optimized, and the Pd^II^ center lies slightly above the mean plane. The (**A**--Pd) quasi-bond lengths (R_Pd__…**A**_ = 2.782 ~ 2.935 Å) between the axial **A** site and Pd^II^ center are much shorter than the corresponding bonds in the other *endo*- and *exo*-Pd tetracoordinates. The optimized structure of *endo*-Pd([9]ane**B**_2_**A**)Cl_2_ complex with a *trans* Cl-acceptor has a tetracoordinate geometry. The distances between the axial **A** site and Pd^II^ center are long (R_Pd__…**A**_ = 3.033~3.190 Å). Our values are in agreement with the geometric structures and the apical (soft **A**…Pd) distances (R_(Pd__…A)_ = 3.087~3.293 Å) seen in the previous crystal studies [37,40,41,42].

The (soft **A**--Pd) quasi-bond in pentacoordinate *endo*-[Pd([9]ane**B**_2_**A**)(**L**-donor)_2_]^2+^ complex causes an isomer of that complex to be more stable than tetracoordinate *endo*-[Pd([9]ane**BAB**)(**L**-donor)_2_]^2+^ complex {ΔE_BAB-B2A_ = 0.10 ~ 0.37 eV for *endo*-[Pd([9]aneN_2_P)(**L**-donor)_2_]^2+^, ΔE_BAB-B2A_ = 0.08 ~ 0.35 eV for *endo*-[Pd([9]aneN_2_S) (**L**-donor)_2_]^2+^}. The relative energy of *endo*-[Pd([9]ane**B**_2_**A**)(**L**-donor)_2_]^2+^ pentacoordinate (ΔE_exo-endo_ = 0.23~0.70 eV) is lower than the corresponding *exo*-type tetracoordinate. Except for these four *endo*-Pd pentacoordinate complexes, the relative energy of *exo*-[Pd([9]ane**BAB**) (**L**-donor)_2_]^2+^ tetracoordinate is lower than that of *exo*-[Pd([9]ane**B**_2_**A**)(**L**-donor)_2_]^2+^ tetracoordinate {ΔE_BAB-B2A_ = −0.70 ~ −0.20 eV for *endo*-Pd([9]ane**BAB**)(**L**-donor)_2_]^2+^}. The relative energy of tetracoordinate [Pd([9]ane**BAB**)Cl_2_ complex is also lower than that of [Pd([9]ane**B**_2_**A**)Cl_2_ {ΔE_BAB-B2A_ = −0.35 ~ −0.18 eV for Pd([9]ane**BAB**)**L**_2_}. Therefore, the axial (soft **A--**Pd) quasi-bond contributes largely to their relative stability. The geometric parameters listed in Table 1 are very similar to the corresponding parameters given in Supplementary Information Tables S2 and S3.

**Table 1:** Optimized average bond distances (Å), average atomic charges (CHelpG, au), and relative energies (eV) of the equilibrium structures of {Pd([9]ane**B**_2_**A**)**L**_2_ and Pd([9]ane**BAB**)**L**_2_} at the B3P86/6-311+G** (lanl2DZ for Pd) level.

Compound	Average distance				Average atomic charge				Relative energy		
	R^a^_Pd-N_	R^a^_Pd-P_	R^b^_Pd__…__N_	R^b^_Pd__…__P_	Q^c^_Pd_	Q^c^_PH3_	Q^d^_N_	Q^d^_P_	ΔE^e^_H-L_	ΔE^f^_BAB-B2A_	ΔE^g^_exo-endo_
*endo*-[Pd([9]aneNPN)(PH_3_)_2_]^2+^	2.195	2.289	2.848		0.348	0.368	−0.151		3.45	0.37	0.00
*exptl*		2.3214^h^	2.298^i^2.3207^i^								
*endo*-[Pd([9]aneN_2_P)(PH_3_)_2_]^2+^	2.142			2.782	0.225	0.412		−0.289	2.68	0.00	0.00
*theo*		2.340^j^			0.598^k^	0.150^k^					
*exptl*		2.3337^i^									
*exo-*[Pd([9]aneNPN)(PH_3_)_2_]^2+^	2.154	2.363	3.504		0.405	0.380	−0.152		3.39	−0.70	−0.37
*exo-*[Pd([9]aneN_2_P)(PH_3_)_2_]^2+^	2.129			3.963	0.263	0.449		−0.018	2.74	0.00	0.70
	R^a^_Pd-N_	R^a^_Pd-S_	R^b^_Pd__…__N_	R^b^_Pd__…__S_	Q^c^_Pd_	Q^c^_PH3_	Q^d^_N_	Q^d^_S_			
*endo*-Pd([9]aneNSN)(PH_3_)_2_]^2+^	2.155	2.359	2.720		0.332	0.377	−0.159		3.40	0.10	0.00
*endo*-[Pd([9]aneN_2_S)(PH_3_)_2_]^2+^	2.138			2.877	0.294	0.447		−0.269	2.67	0.00	0.00
*exo-*[Pd([9]aneNSN)(PH_3_)_2_]^2+^	2.135	2.382	3.602		0.435	0.366	−0.112		2.87	−0.25	−0.03
*exo-*[Pd([9]aneN_2_S)(PH_3_)_2_]^2+^	2.127			3.954	0.343	0.444		−0.033	2.42	0.00	0.32
	R^a^_Pd-N_	R^a^_Pd-P_	R^b^_Pd__…__N_	R^b^_Pd__…__P_	Q^c^_Pd_	Q^c^_P(CH3)3_	Q^d^_N_	Q^d^_P_	ΔE^e^_H-L_	ΔE^f^_BAB-B2A_	ΔE^g^_exo-endo_
*endo*-[Pd([9]aneNPN){P(CH_3_)_3_}_2_]^2+^	2.214	2.324	3.076		0.464	0.425	−0.133		4.01	0.35	0.00
*endo*-[Pd([9]aneN_2_P){P(CH_3_)_3_}_2_]^2+^	2.195			2.843	0.380	0.389		−0.416	3.51	0.00	0.00
*exo-*[Pd([9]aneNPN){P(CH_3_)_3_}_2_]^2+^	2.188	2.368	3.678		0.575	0.439	0.048		3.85	−0.51	−0.28
*exo-*[Pd([9]aneN_2_P){P(CH_3_)_3_}_2_]^2+^	2.178			4.049	0.414	0.372		−0.061	3.52	0.00	0.58
	R^a^_Pd-N_	R^a^_Pd-S_	R^b^_Pd__…__N_	R^b^_Pd__…__S_	Q^c^_Pd_	Q^c^_P(CH3)3_	Q^d^_N_	Q^d^_S_			
*endo*-Pd([9]aneNSN){P(CH_3_)_3_}_2_]^2+^	2.185	2.402	2.920		0.409	-0.392	−0.118		3.88	0.08	0.00
*endo*-[Pd([9]aneN_2_S){P(CH_3_)_3_}_2_]^2+^	2.194			2.935	0.353	-0.396		−0.300	3.50	0.00	0.00
*exo-*[Pd([9]aneNSN){P(CH_3_)_3_}_2_]^2+^	2.179	2.420	3.705		0.434	-0.352	0.129		3.55	−0.20	−0.05
*exo-*[Pd([9]aneN_2_S){P(CH_3_)_3_}_2_]^2+^	2.176			4.037	0.458	-0.412		−0.107	3.19	0.00	0.23
	R^a^_Pd-N_	R^a^_Pd-P_	R^b^_Pd__…__N_	R^b^_Pd__…__P_	Q^c^_Pd_	Q^c^_Cl_	Q^d^_N_	Q^d^_P_	ΔE^e^_H-L_	ΔE^f^_BAB-B2A_	ΔE^g^_exo-endo_
*endo*-Pd([9]aneNPN)Cl_2_	2.113	2.225	3.040		0.801	−0.746	−0.077		4.04	−0.28	0.00
*exptl*	2.115^l^	2.3337^i^									
	2.117^l^										
*endo*-Pd([9]aneN_2_P)Cl_2_	2.088			3.033	0.720	−0.740		−0.357	3.84	0.00	0.00
*theo*	2.101^k^				0.628^k^	−0.516^k^					
	2.049^k^										
*exptl*	2.0410^i^										
	2.0354^i^										
*exo-*Pd([9]aneNPN)Cl_2_	2.106	2.346	3.471		0.920	−0.762	−0.086		4.08	−0.35	−0.13
*exo-*Pd([9]aneN_2_P)Cl_2_	2.095			3.868	0.695	−0.708		−0.152	4.03	0.00	−0.06
	R^a^_Pd-N_	R^a^_Pd-S_	R^b^_Pd__…__N_	R^b^_Pd__…__S_	Q^c^_Pd_	Q^c^_Cl_	Q^d^_N_	Q^d^_S_			
*endo*-Pd([9]aneNSN)Cl_2_	2.094	2.324	2.923		0.771	−0.747	−0.073		3.73	-0.18	0.00
*exptl*	2.124^m^	2.2492^m^	2.638^m^								
		2.332^n^									
*endo*-Pd([9]aneN_2_S)Cl_2_	2.091			3.190	0.609	−0.722		−0.304	3.77	0.00	0.00
*exptl*	2.077^m^			3.0865^m^ 3.293^o^							
	2.117^o^										
	2.144^o^										
*exo-*Pd([9]aneNSN)Cl_2_	2.094	2.329	3.465		0.787	−0.727	0.076		3.85	−0.21	−0.13
*exptl*	2.068^p^	2.2685^m^	3.499^p^								
	2.050^p^	2.317^q^									
*exo-*Pd([9]aneN_2_S)Cl_2_	2.094			3.854	0.774	−0.724		−0.178	4.04	0.00	−0.09

^a^Bond length between the Pd^II^ center and equatorial binding site of the tridentate ligand; ^b^Bond length between the Pd^II^ center and axial binding site of the tridentate; ^c^Atomic charges of the Pd^II^ center and the binding atom of *trans* **L**-donor; ^d^Atomic charge of the apical binding atom; ^e^Energy gap between HOMO and LUMO; ^f^Relative energy gap between Pd([9]ane**BAB**)**L**_2_ and Pd([9]ane**B**_2_**A**)**L**_2_; ^g^Energy gap between *exo*- and *endo*-type structures; ^h^Ref. [23]; ^i^Ref. [42]; ^j^Ref. [17]; ^k^Ref. [44]; ^l^Ref. [18]; ^m^Ref. [40]; ^n^Ref. [53]; ^o^Ref. [41]; ^p^Ref. [37]; ^q^Ref. [54].

In previous studies [18,19,20,21,22,23,24,25,26,27,28,29,30,31,32,33,34,35,36], unusual penta-, hexa-, and octacoordinate Pd complexes with some apical (**A**-Pd) bonds were synthesized under some restrictions such as electronic effect of the strong σ-donor in an electron-rich *trans* **L**-ligand and steric effect of the bulky ligand. In the Pd-mediated cross-coupling reactions of an allylic group, the geometry of the (allyl)Pd**L**_2_ crystals was observed to be distorted penta- and hexacoordinate structures [18,19,20,21,22,23,24]. Three carbons of an allylic group (η^3^-C_3_H_5_) are axially coordinated to the Pd center of the Pd^(0)^**L**_n_ precursor. The average distances of the apical (allyl-Pd) bond are short (R_C-Pd_ = 2.101~2.253 Å) [18,19,22,23]. Interestingly, in the aza-macrocyclic Pd^(0)^(C_10_H_16_N_2_Me_2_) complexes [33,34,35] and macrocyclic Pd**L** complexes where **L** = *1,4a,5,8a-*tetra-hydronaphthalene-2,6-dione [36], the six and eight coordination bonds of the 3~4 olefin units with Pd were formed by the steric effect of the bulky ligand. Due to the steric hindrance, none of these three carbons of the olefin units was positioned at the edge of the formal geometry (*i.e.*, the trigonal bipyramid or square pyramid) [18,19,20,21,22,23,24,25,26,27,28,29]. Furthermore, in the (olefin)Pd**L**_2_ complex with an apical olefin-Pd bond, various (olefin)Pd**L**_2_ intermediates were observed during the Pd-mediated cross-coupling reactions [20,25,26,30]. However, in [Pd([9]ane**N**_3_)_2_]^2+^ and [Pd([9]ane**N**_3_)_2_(H[9]ane**N**_3_)_2_]^3+^ complexes without geometric constraints, the structures with an apical (soft **A**…Pd) interaction were not observed [40].

**Figure 2:**
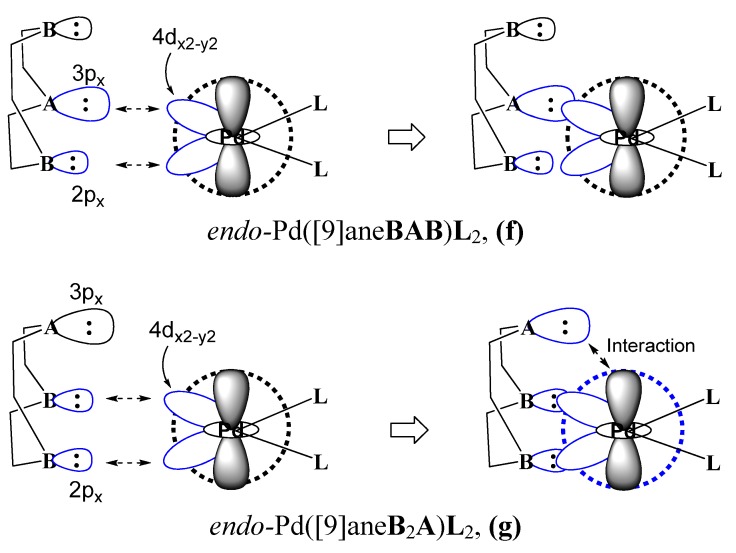
The equatorial coordination bonds of the **A** (or **B**) site to the 4d_x_^2^_-y_^2^-orbital and the axial (**A**…Pd) interaction in the *endo*-Pd([9]ane**B**_2_**A**)**L**_2_ complexes.

As shown in Figure 2, when the soft **A** and hard **B** binding sites simultaneously coordinate to the 4d_x2-y2_ orbital, the large σ-donor of the **A** site overlaps more with the 4d_x2-y2_ orbital than the small σ-donor of **B**. With increasing atomic size (P:107 pm ≅ S:105 pm >> N:71 pm) [52], the larger σ-orbital of the soft **A** site in [9]ane**BAB** first interacts with the 4d_x2-y2_ orbital on the square-planar plane. In *endo*-Pd([9]ane**BAB**)**L**_2_ of **(f)** of Figure 2, due to its atomic size, the bond length (r_Pd__-__P_ = 2.324 ~ 2.225 Å and r_P__d-S_ = 2.402 ~ 2.324 Å) of the equatorial (A-Pd) bond is longer than the corresponding (N-Pd) bond (r_P__d-N_ = 2.214 ~ 2.094 Å). Two equatorial bond lengths (r_P__d-A_, r_P__d-B_) are unsymmetrical in nature. The axial distance between the axial **N** site and Pd^II^ center is long (r_P__d-N_ = 2.720 ~ 3.076 Å). As shown in **(g)** of Figure 2, in the *endo*-[Pd([9]ane**B**_2_**A**)(**L**-donor)_2_]^2+^ complex, the average distance between the equatorial **N** site and Pd^II^ center is short (R_P__d-**N**_ = 2.138 ~ 2.195 Å). Because both the equatorial (**N**-Pd) bonds are short, the large σ-donor of the apical **A** site closely approaches to the Pd center. Thus, the large σ-donor of the soft **A** site can interact with the low-lying unoccupied a_1g_(5s)-orbital of Pd**L**_2_ complex. 

The ratio of products in Pd-catalyzed cross-coupling reactions was experimentally determined by the strength of the σ-donor ligand (e.g., *n*-Bu_3_N or an acetate anion) [6,30,31]. As the softness (P ≅ S > N) [47] and basicity (N > P > S) [6,26] of the binding site increases, the coordination bond of the soft **A** site to the 4d_x2-y2_-orbital was more preferred than that of the hard **B** site. That is, the σ-donor of the soft **A** site demonstrates a stronger overlap on the 4d_x2-y2_ orbital than the hard **B** site. As shown in Table 1, *exo-*[Pd([9]ane**BAB**)(**L**-donor)_2_]^2+^ and Pd([9]ane**BAB**)Cl_2_ complexes with an equatorial (**A**-Pd) bond are more stable than the corresponding *exo-*[Pd([9]ane**B_2_A**)(**L**-donor)_2_]^2+^ and Pd([9]ane**B**_2_**A**)Cl_2_ isomers without the equatorial (**A**-Pd) bond, respectively. As described in Supplementary Information Figure **S1** and Table **S1**, *endo-*[Pd([9]ane**PNP**)(**L**-donor)_2_]^2+^ could not be optimized. By strong softness and basicity of the binding **A** site, the relative energy of tetracoordinate *endo-*[Pd([9]ane**A_2_B**)(**L**-donor)_2_]^2+^ complex with two equatorial (**A**-Pd) bonds is lower than that of pentacoordinate *endo-*[Pd([9]ane**ABA**)(**L**-donor)_2_]^2+^ complex with an axial (**A**-Pd) bond {ΔE_BAB-B2A_ = −0.01 ~ −0.02 eV for *endo*-Pd([9]aneS_2_N)(**L**-donor)_2_]^2+^}. However, in the *endo*-[Pd([9]ane**B**_2_**A**)(**L**-donor)_2_]^2+^ complex with an axial fifth (**A**--Pd) quasi-bond, the pentacoordinate Pd complex is more stable than the tetracoordinate *endo*-[Pd([9]ane**BAB**)(**L**-donor)_2_]^2+^ complexes. Therefore, in case of interaction of [9]ane**B**_2_**A** to Pd**L**_2_, the relative stability of the Pd complexes and the selective formation of the coordination bond may depend on the ***strength*** of the hardness/softness and basicity of **A** (or **B**) and the ***position*** of the equatorial (or apical) binding atoms, and the ***existence*** of an axial (**A**--Pd) quasi-bond [6,26,30,31,47,55].

In some Pd^II^ complexes with terminal amino derivatives [37,38,39,40,41,42], both apical soft **S**…Pd and hard **N**…Pd interactions were observed under the strong strain of a polymeric side chain. In particular, the apical (hard **N**…Pd) distances (r_Pd__…__N_ = 2.523 ~ 2.638 Å) [40] are slightly shorter as explained by an axial σ*…d_z2_ interaction between an antibonding orbital of the apical N site and the filled d_z2_-orbital [42]. Meanwhile, in the ligand exchange processes of the Pd complexes [46,47], an axial water…Pd interaction was also observed. The mechanism of the hydration reactions is described by two models: axial H_2_O…d_z_^2^-orbital and OH_2_…d_z_^2^-orbital interactions with the former being rarely formed due to electrostatic repulsions. In studies by Kozelka *et al*. [56], both the axial O_2_H…Pt and H_2_O…Pt interactions in the Pt complex were explained by an electrostatic attraction between the dispersion components. Until now, the axial **L**…d^8^-metal interaction between the axial **L-**ligand and d^8^-metal have not been explained in detail using an orbital interaction. 

**Figure 3:**
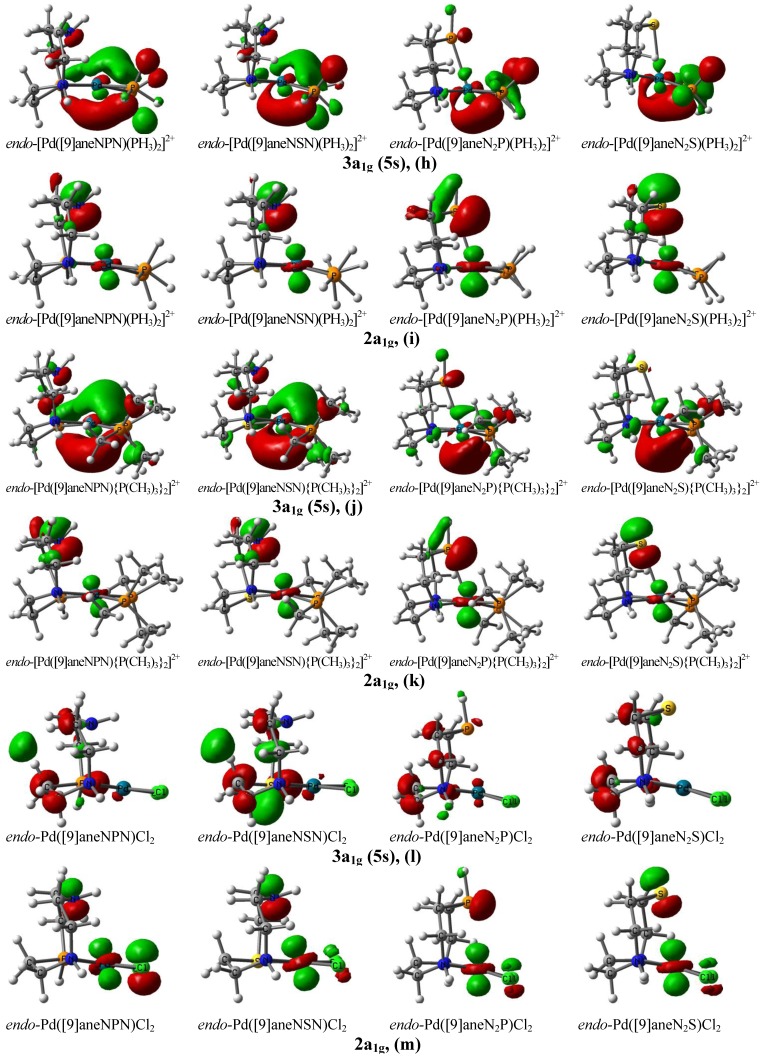
The 3a_1g_(5s)-orbital [(**h**), (**j**), (**l**)] and 2a_1g_-orbital [(**i**), (**k**), (**m**)] of *endo*-Pd([9]ane**B**_2_**A**)**L**_2_ and *endo*-Pd([9]ane**BAB**)**L**_2_ complexes calculated at the B3P86/6-311+G** (lanl2DZ for Pd) level, respectively.

The 3a_1g_(5s)-orbital [(**h**), (**j**), (**l**)] and the 2a_1g_-orbital [(**i**), (**k**), (**m**)] of *endo*-Pd complexes are illustrated in Figure 3. As shown in **(h)**, **(j)**, and **(l)** of Figure 3, the orbital shape of the 3a_1g_(5s)-orbital of the *endo*-Pd([9]ane**B**_2_**A**)**L**_2_ complexes is largely varied by the donating (or withdrawing) property of the *trans* **L**-ligand. Due to the electron donating property of *trans* **L**-donor, its electron density moves to the Pd^II^ center, and then the increased electron density at the Pd^II^ center is transferred to the low-lying unoccupied 3a_1g_(5s)-orbital. The orbital lobe of the 3a_1g_(5s)-orbital around the Pd^II^ center is huge [as shown in tetracoordinate *endo*-[Pd([9]ane**BAB**)(**L**-donor)_2_]^2+^ complex of **(h)** and **(j)** of Figure 3]. This huge lobe of the 3a_1g_(5s)-orbital can interact with the σ-donor of the soft **A** site [or filled π-donor orbital of substrates]. In the *endo*-[Pd([9]ane**B**_2_**A**)(**L**-donor)_2_]^2+^ complex with an apical (**A**--Pd) quasi-bond [**(h)** and **(j)** of Figure 3], the shape of the 3a_1g_(5s)-orbital is not symmetric. The partially unfilled and lowered a_1g_(5s)-orbital strongly interacts with the large σ-donor of **A** to make the apical (**A**--Pd) quasi-bond. The upper part of the a_1g_(5s)-orbital lobe is used for the apical (**A**…Pd) interaction. Meanwhile, in the *endo*-[Pd([9]ane**BAB**)(**L**-donor)_2_]^2+^ complex without the apical (**A**--Pd) quasi-bond [**(h)** and **(j)** of Figure 3], the orbital lobe of the 3a_1g_(5s)-orbital is huge and symmetric. The 3a_1g_(5s)-orbital of *endo*-[Pd([9]ane**BAB**)(**L**-donor)_2_]^2+^ complex is quite different from that of *endo*-[Pd([9]ane**B**_2_**A**)(**L**-donor)_2_]^2+^. As shown in **(l)** of Figure 3 with *trans* Cl-acceptor, the lobe size of the 3a_1g_(5s)-orbital is very small. No interaction between the σ-donor of the soft **A** site and the 3a_1g_(5s)-orbital is formed. The orbital shapes of the 3a_1g_(5s)-orbital and the 2a_1g_-orbital are very similar to those given in Supplementary Information Figures **S2** and **S3**. As listed in Table 1, the atomic charge of Pd in [Pd([9]ane**B**_2_**A**)(**L**-donor)_2_]^2+^ complex is more negative than that of Pd in Pd([9]ane**B**_2_**A**)Cl_2_ complex.

**Figure 4:**
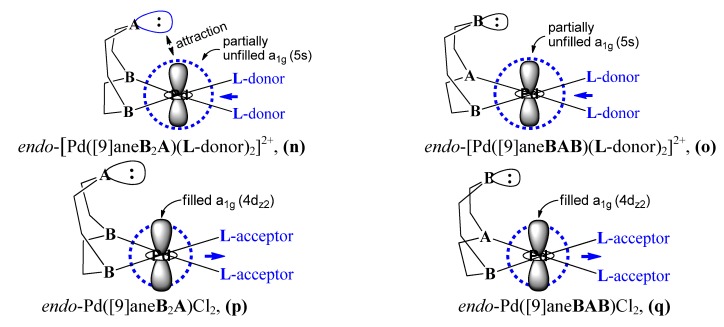
The axial orbital interaction between the σ-donor of the soft **A** site (or hard **B** site) and the partially unfilled a_1g_(5s)-orbital is influenced by the electronic property of the *trans* **L-**ligand and the size of the σ-donor of **A** (or **B**).

Based on Figure 3, orbital interaction between the σ-donor of the apical soft **A** (or hard **B**) site and the partially unfilled a_1g_(5s)-orbital of Pd^II^ center as well as the direction for the electron transfer of *trans* **L**-ligand (electron-donor or acceptor) are schematically depicted in Figure 4. In *endo*-[Pd([9]ane**B**_2_**A**)(**L**-donor)_2_]^2+^ and *endo*-[Pd([9]ane**BAB**)(**L**-donor)_2_]^2+^ complexes, the electron density of the unfilled a_1g_(5s)-orbital of Pd^II^ center is greatly increased by the strong electron-donating property of the *trans* **L-**donor. The spatial distribution of electron density of the a_1g_(5s)-orbital is larger than that of the a_1g_(4d_z_^2^)-orbital, thus positioning the lobe of a_1g_(5s)-orbital at the outer space of the a_1g_(4d_z_^2^)-orbital. As represented in **(n)** of Figure 4, the soft σ-donor of the axial **A** site (or substrate of Lewis base) first interacts with the partially unoccupied a_1g_(5s)-orbital (or Pd-complex of Lewis acid). A fifth apical (soft **A**--Pd) quasi-bond is formed as shown in Figure 1. In **(o)** of Figure 4, the size of σ-donor in an axial **B** site is small [covalent radius of the N atom: 71 pm [52]]. In the regular Pd tetracoordinate, the axially hard **N** atom cannot easily interact with the Pd center. Meanwhile, in *endo*-Pd([9]ane**B**_2_**A**)Cl_2_ and *endo*-Pd([9]ane**BAB**)Cl_2_ complexes with a *trans* Cl-acceptor, the filled a_1g_(4d_z_^2^)-orbital of Pd^II^ center lies spatially outside than that of the a_1g_(5s)-orbital. There is no interaction between the soft σ-donor of **A** and the filled a_1g_(4d_z_^2^)-orbital. 

Experiments to analyze the electronic effect of a donating (or withdrawing) Z group were performed by some research groups [30,31]. The results of these experiments indicated that the rate of catalytic activity of the neutral Pd^0^(dba-*n,n'*-Z)_2_ precursor is greatly dependent on the electronic property (donor or acceptor) of the bulky dba-*n,n’*-Z ligand. Owing to the increase in the strength of electron donating dba-*n,n’*-Z ligand, the overall rate of the oxidative addition of phenyl iodide to Pd^0^(dba-*n,n’*-Z)_2_ precusor is faster than that of the Pd^0^ complex with an electron withdrawing Z group. In particular, the rate of oxidative addition of aryl halide to Pd^0^**L**_n_ depends on the concentration of the active Pd^0^**L**_n_ precursor with a strong electron-donating *trans* **L**-ligand. Scrivanti *et al.* [38,39] found that the rate of oxidative addition of an aryl halide to the (iminophosphine)Pd^0^(η^2^-olefin) complex also increased. The increase in reaction rate is explained by the catalyst stability of the moderate π-accepting ligand. These experimental results [31,39] showing an increased rate in the catalytic activity may have originated from the electronic property of a strong electron-donating *trans* **L**-ligand in Pd^0^**L**_n_. Based on Figure 3 and Figure 4, in the oxidative addition of ArX to Pd^0^**L**_n_, the donating π-orbital of the aryl halide can interact with the low-lying unfilled a_2u_(5p)-orbital of Pd. Thus, an a_2u_(5p)…π-orbital interaction between the low-lying unfilled a_2u_(5p)-orbital of Pd and the filled π-orbital of ArX can take place. These experimental results can be understood with the help of results shown in Figure 3.

**Figure 5:**
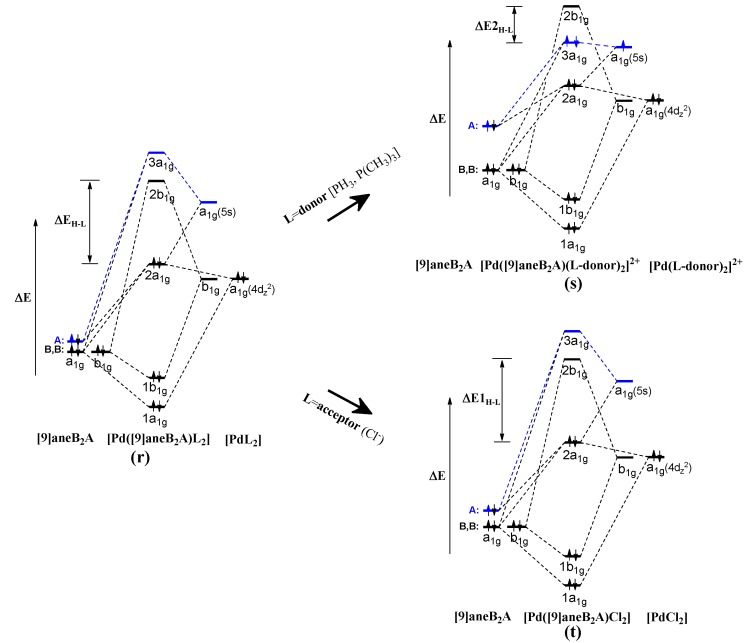
The variation in the orbital energy levels of *endo*-Pd([9]ane**B**_2_**A**)**L**_2_ complex, showing the coordination bonds of [9]ane**B**_2_**A** to [Pd(**L**-donor)_2_]^2+^ and [PdCl_2_]. In *endo*-[Pd([9]ane**B_2_A**)(**L**-donor)_2_]^2+^ complex **(r)**, the separated configuration of two electron spins filled in the 3a_1g_-orbital indicates the (**A**--Pd) quasi-bond.

The orbital energy levels for the orbital interaction associated with the coordination bond of [9]ane**B**_2_**A** to Pd**L**_2_ are drawn in Figure 5. As shown in **(r)** of Figure 5, there is a large gap in the energy level between the **A** site of [9]ane**B**_2_**A** and the a_1g_(5s)-orbital of Pd**L**_2_. Therefore, the σ-orbital of **A** cannot easily interact with the unoccupied a_1g_(5s)-orbital. Meanwhile, as shown in **(s)** of Figure 5, the energy gap between the axial **A** site and a_1g_(5s)-orbital is largely reduced. In the *endo*-[Pd([9]ane**BAB**)(**L**-donor)_2_]^2+^ complex as shown in **(h)** and **(j)** of Figure 3, the unoccupied 3a_1g_(5s)-orbital is partially filled by the electron density transfer from *trans* **L-**donor and ***the level of the partially filled 3a_1g_(5s)-orbital decreases.*** In *endo*-[Pd([9]ane**B**_2_**A**)(**L**-donor)_2_]^2+^ complex as shown in **(i)** and **(k)** of Figure 3, the huge σ-orbital of the soft **A** site can overlap with the partially filled 3a_1g_(5s)-orbital and then ***the energy level of the A site is increased*** by the [σ-donor ↔ 3a_1g_(5s)] overlap. Therefore, the electron density of increased energy level in σ-orbital of **A** can share with that of the decreased energy level of 3a_1g_(5s)-orbital. The unfilled 3a_1g_-molecular orbital is occupied and the energy level is largely lowered as HOMO. The energy difference between the HOMO and LUMO is also reduced {**Δ**E2_H-L_ = 2.67~3.51 eV for *endo*-[Pd([9]ane**B**_2_**A**)(**L**-donor)_2_]^2+^}. As shown in **(t)** of Figure 5, the orbital energy levels are similar to that in **(r)** of Figure 5. The σ-orbital cannot interact with the 3a_1g_(5s)-orbital and the energy gap between the HOMO and LUMO is large {ΔE1_H-L_ = 3.77 and 3.84 eV for *endo*-Pd([9]ane**B**_2_**A**)Cl_2_}.

Similar to the interaction suggested above [σ-donor…unfilled a_1g_(5s)], the fifth [sixth, eighth] axial olefin-Pd coordination bond in the oxidative addition of olefin to Pd^(0)^**L**_n_ can be formed by an axial π-donor…unfilled a_2u_(5p_x,y_) interaction between the π-donor of olefin and an unfilled a_2u_(5p_x,y_)-orbital of Pd. The electron density transfer from the *trans* **L**-donor to the unfilled a_2u_(5p_x,y_)-orbital makes it partially occupied, and thus the energy level of the partially unfilled a_2u_(5p_x,y_)-orbital is lowered. Consequently, the π-donor electron-rich substrates such as olefin can interact with the partially unfilled and lowered a_2u_(5p_x,y_)-orbital of Pd. In the Pd-mediated cross-coupling reactions, the results of this study can describe the mechanism for the formation of the apical σ-donor…unfilled a_1g_(5s) and π-donor…unfilled a_2u_(5p_x,y_) interactions in ArPd**L**_n_X. Furthermore, in d^9^-electron systems such as [Cu(NH_3_)_4_(H_2_O)_2_]^2+^ [57,58], a distorted octahedron with two water molecules at a longer distance than four ammonias is formed *via* the long range interactions. The vertical Cu-OH_2_ bond length (R_Cu-O_ = 2.204 Å) is longer than that (R_Cu-N_ = 1.933 Å) of the equatorial Cu-NH_3_ bond [55]. The two longer Cu-OH_2_ bonds along the z-axis are explained by the interaction between the half unoccupied 3d_z2_-orbital of Cu^II^ and the filled σ-orbital of the oxygen atom in water.

**Figure 6:**
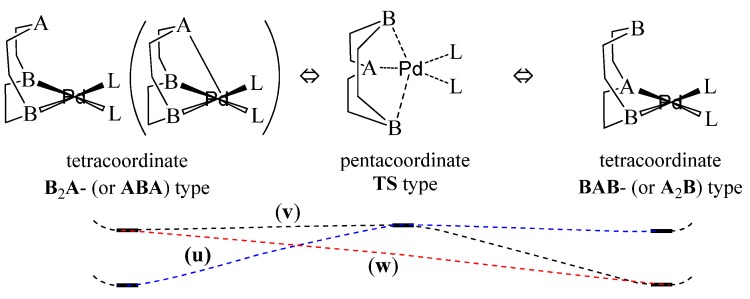
The variation of the structural configuration from *endo*-Pd([9]ane**B**_2_**A**)**L**_2_ {or *endo*-Pd([9]ane**ABA**)**L**_2_} complex to *endo*-Pd([9]ane**BAB**)**L**_2_ {or *endo*-Pd([9]ane**A**_2_**B**)**L**_2_} complex, including the relative energy level (**A**:P, S; **B**:N). (**u**) is a reaction path from the pentacoordinate *endo*-[Pd([9]ane**B**_2_**A**)(**L**-donor)_2_]^2+^ complex with an axial (soft **A**--Pd) quasi-bond to *endo*-[Pd([9]ane**BAB**)(**L**-donor)_2_]^2+^. (**v**) is a reaction path from the tetracoordinate *endo*-Pd([9]ane**B**_2_**A**)(**L**-acceptor)_2_ complex with a *trans* **L**-acceptor to *endo*-Pd([9]ane**BAB**)(**L**-acceptor)_2_. (**w**) is a reaction path from the *endo-*Pd([9]ane**PNP**)(**L**-donor)_2_ complex to *endo-*Pd([9]ane**P**_2_**N**)(**L**-donor)_2_ without the transition energy barrier.

The relative energies through the structural variation from *endo*-Pd([9]ane**B**_2_**A**)**L**_2_ complex to *endo*-Pd([9]ane**BAB**)**L**_2_ are represented in Figure 6. In the **(u)** reaction path, the relative energy level of *endo*-[Pd([9]ane**B**_2_**A**)(**L**-donor)_2_]^2+^ complex with the fifth (**A**--Pd) quasi-bond is lower than that of *endo*-[Pd([9]ane**BAB**)(**L**-donor)_2_]^2+^ {ΔE_BAB-B2A_ = −0.37, −0.10, −0.35, and −0.08 eV for *endo*-[Pd([9]ane**B**_2_**A**)(**L**-donor)_2_]^2+^}. The axial (**A**--Pd) quasi-bond greatly contributes to the relative stability of the pentacoordinate *endo*-[Pd([9]ane**B**_2_**A**)(**L**-donor)_2_]^2+^ complexes. In the **(v)** reaction path, the relative energy level of *endo*-Pd([9]ane**BAB**)Cl_2_ {*endo-*Pd([9]ane**A**_2_**B**)Cl_2_} complex is more stable than that of *endo*-Pd([9]ane**B**_2_**A**)Cl_2_ {*endo-*Pd([9]ane**ABA**)Cl_2_} complex (ΔE = −0.18~−0.28 eV for *endo*-Pd([9]ane**BAB**)Cl_2_). Due to the relative stability between the axial and equatorial (**A**-Pd) bonds, the Pd([9]ane**ABA**)**L**_2_ and Pd([9]ane**A_2_B**)**L**_2_ structures with one or two equatorial (**A**-Pd) bonds are more stable than the Pd([9]ane**B**_2_**A**)**L**_2_ and Pd([9]ane**BAB**)**L**_2_ structures, respectively (as shown in Table 1 and Supplementary Information Table **S1**). In particular, in the **(w)** reaction path, the geometric configuration of *endo-*[Pd([9]ane**PNP**)(**L**-donor)_2_]^2+^ automatically optimizes to the *endo-*[Pd([9]ane**P_2_N**)(**L**-donor)_2_]^2+^ structure without the transition energy barrier. {*endo-*[Pd([9]ane**PNP**)(**L**-donor)_2_]^2+^ could not be optimized.} Therefore, the relative stability and configurational and conformational changes (such as axial-equatorial coordination and *cis-trans* isomerization) of the RPd^II^(**L**)_n_X intermediates are largely influenced by the axial (soft **A**…Pd) interaction, relative Pd affinity of the soft **A** atom, and low potential energy barrier. 

## 4. Conclusions

We investigated the geometric structures and relative stabilities of Pd([9]ane**B**_2_**A**)**L**_2_ complexes {Pd([9]ane**BAB**)**L**_2_, *endo*-Pd([9]ane**A**_2_**B**)**L**_2_, *endo*-Pd([9]ane**ABA**)**L**_2_}, the selective orbital interaction for an axial or equatorial (**A**…Pd) coordination of [9]ane**B**_2_**A** with Pd**L**_2_, and the electronic effects of the soft **A/**hard **B** donors and the donating/withdrawing *trans* **L**-ligand. The *endo*-[Pd([9]ane**B**_2_**A**)(**L**-donor)_2_]^2+^ complex with an axial (soft **A**--Pd) quasi-bond was optimized as a pentacoordinate geometry, while the other Pd-complexes were optimized as tetracoordinate structures. The relative energy of the pentacoordinate Pd complexes is lower than that of the corresponding *endo*-Pd tetracoordinates. Among the Pd tetracoordinated species, the Pd([9]ane**BAB**)**L**_2_ structure with an equatorial (soft **A**-Pd) bond is more stable than the Pd([9]ane**B**_2_**A**)**L**_2_ type with both the equatorial (hard **B**-Pd) bonds. Furthermore, the *endo-*[Pd([9]ane**A_2_B**)(**L**-donor)_2_]^2+^ tetracoordinated with two equatorial (**A**-Pd) bonds is more stable than pentacoordinated *endo-*[Pd([9]ane**ABA**)(**L**-donor)_2_]^2+^. And the *endo*-[Pd([9]ane**PN**P)(**L**-donor)_2_]^2+^ type of structures is automatically optimized to the stable *endo-*[Pd([9]ane**P_2_N**)(**L**-donor)_2_]^2+^ type of structures. The relative stability of the Pd-complexes and the equatorial or axial selectivity of the soft **A**/hard **B** donors to Pd^II^ center are dependent upon its softness, basicity, and electronic effects. 

In the Pd-catalyzed coupling reactions, the Pd**L**_n_ intermediates are activated by an electron-rich *trans* **L**-donor possessing a strong Lewis base character. The strong Lewis base character of the *trans* **L**-donor allows its electron density to transfer to the low-lying unoccupied orbitals [a_1g_(5s) or a_2u_(5p)] of Pd and the energy level of the partially unfilled a_1g_(5s)-orbital of Pd is decreased. Therefore, an axially fifth substrate…Pd**L**_2_ interaction between the σ(or π)-donor of substrates (such as hemilabile multidentates, olefinic halides, and solvents) and the Pd**L**_2_ precursor should occur energetically, resulting in the electronic and steric effects of the bulky and electron-rich ligand. Because of the low transition barrier and an axial substrate…Pd**L**_2_ interaction, the configurational and conformational changes of RPd^II^(**L**)_n_X can easily take place (e.g., axial-equatorial and *cis-trans* exchanges and interconversions in RPd^II^(**L**)_n_X intermediates). Consequently, the geometric structure and relative stability of the Pd complexes are influenced by the relative strength of the axial or equatorial (soft **A**…Pd) interaction, donating electron property of the *trans* **L**-ligand, and relative Pd affinity of the **A** and **B** donors. The mechanisms for orbital interaction and electron density transfer proposed in this study can be considerably valuable in the design of useful Pd-catalyst and synthetic applications for the Pd-catalyzed cross-couplings of substrates.

## Supplementary Materials

Supplementary materials can be accessed at: http://www.mdpi.com/1420-3049/18/10/12687/s1.
